# A retrospective clinical analysis of pediatric mediastinal space-occupying lesions in a Chinese children’s hospital from 2015 to 2024

**DOI:** 10.3389/fonc.2026.1788203

**Published:** 2026-03-25

**Authors:** Mengjiao Zhao, Li Song, Hongwei Zhang, Ning Xue, Shuai Chen, Qingwei Guo

**Affiliations:** 1 Department of Pulmonary Intervention, Jinan Children’s Hospital, Jinan, China; 2 Department of Hematology and Oncology, Jinan Children’s Hospital, Jinan, China; 3 Department of Neurology, Jinan Children’s Hospital, Jinan, China; 4 Department of Gastroenterology, Jinan Children’s Hospital, Jinan, China; 5 Department of Pediatric Surgery, Jinan Children’s Hospital, Jinan, China; 6 Department of Child Health, and Department of Hematology and Oncology, Jinan Children’s Hospital, Jinan, China

**Keywords:** pediatric, mediastinal space‑occupying lesions, differential diagnosis, retrospective analysis, age distribution, compartment localization

## Abstract

**Background:**

Pediatric mediastinal space-occupying lesions (MSOLs) encompass a wide spectrum of conditions, including benign and malignant, infectious and reactive, as well as congenital and acquired. The differential diagnosis of pediatric MSOLs remains a clinical challenge. This single-center study aimed to analyze relevant clinical features of MSOLs and to improve the accuracy of their differential diagnosis.

**Methods:**

A retrospective analysis was conducted of MSOLs diagnosed over a 10-year period. Information on etiology, demographic characteristics, lesion localization, clinical manifestations, and diagnostic approach was collected.

**Results:**

We reviewed 461 cases of pediatric MSOLs from a single institution. The most common type was lymphoma, followed by neurogenic tumors, congenital diaphragmatic hernia, foregut duplication cysts, and infectious diseases. Etiology varied significantly across age groups. Hiatus hernia was the most common lesion in infants (0–12 months), while lymphoblastic lymphoma predominated in children (12–180 months). Lesion distribution also differed among mediastinal compartments: T-lymphoblastic lymphoma was most frequent in the prevascular compartment, hiatus hernia in the visceral compartment, and neuroblastoma in the paravertebral compartment. Forty patients (8.7%) were asymptomatic. Among symptomatic patients, common presentations included cough, chest tightness, fever, nausea/vomiting, chest pain, and facial or neck edema. Malignancies were predominantly found in the prevascular and paravertebral compartments with chest symptoms, whereas non-neoplastic lesions mainly involved the visceral compartment and presented with gastrointestinal symptoms or asymptomatic. Diagnostic approaches included non-invasive imaging and biopsy. Definitive diagnoses were established by imaging alone in 139 cases (30.2%); the remaining patients underwent image-guided or surgical biopsies.

**Conclusion:**

This study reveals a diverse, age-dependent spectrum of pediatric MSOLs. Accurate diagnosis relies on a combination of clinical presentation, compartment-specific localization, and a multidisciplinary approach utilizing both advanced imaging and image-guided or surgical biopsies, which were demonstrated to be safe and effective. These findings can aid significantly in the differential diagnosis and management of pediatric MSOLs.

## Introduction

**Figure 1:**
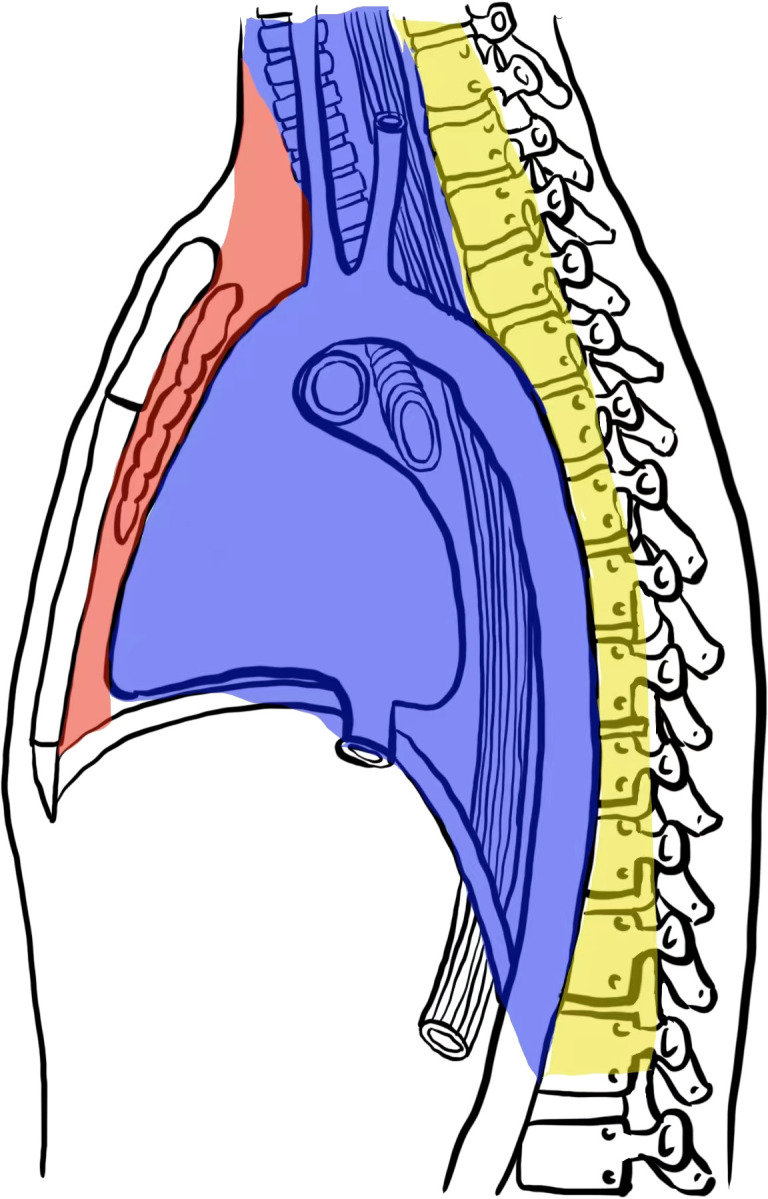
Diagram illustrates the ITMIG based depiction of the prevascular (pink), visceral (purple), and paravertebral (yellow) mediastinal compartments.

The mediastinum is a complex anatomical region between the lungs that contains vital luminal structures (e.g., heart, great vessels, thoracic duct, trachea/bronchi, and esophagus). Dividing the mediastinum into specific compartments has long aided in the identification, characterization, and management of mediastinal abnormalities. Numerous classification systems have been developed and adopted to varying extents by anatomists, surgeons, and radiologists. Recently, the International Thymic Malignancy Interest Group (ITMIG) proposed a new clinical classification of the mediastinum (1, 2), which divides it into prevascular (anterior), visceral (middle), and paravertebral (posterior) compartments (**Figure 1**).

The mediastinum is the most frequent site of space-occupying lesions in children (3). Mediastinal space-occupying lesions (MSOLs) may arise from structures normally located in or passing through the mediastinum during development, as well as from metastases of malignancies originating elsewhere. MSOLs encompass a broad histopathological and radiological spectrum—including benign and malignant, infectious and reactive entities—and can occur at any age. The most common MSOLs in children are neoplastic, infectious, and congenital lesions (4). Frequent neoplastic lesions include lymphoma, neurogenic tumors, and germ cell tumors; congenital lesions include foregut duplication cyst (FDC), congenital diaphragmatic hernia (CDH), and vascular anomalies; and infectious lesions include mediastinal abscess (MA), fibrosing mediastinitis (FM), and Mycobacterium tuberculosis (MTB). Each disease type tends to occur in a characteristic mediastinal compartment.

Children with MSOLs may be asymptomatic or present with symptoms when the lesion compresses or invades adjacent structures (5–9). Common presentations include chest complaints (e.g., dyspnea, cough, chest pain), neck manifestations (e.g., palpable mass, dysphagia), and neurologic signs secondary to spinal cord compression. Superior vena cava syndrome may also occur. Some MSOLs are life-threatening; delayed diagnosis and treatment are associated with poor prognosis. Prompt and accurate diagnosis is therefore critical to guide timely management.

However, studies on pediatric MSOLs remain limited, and differential diagnosis continues to pose a clinical challenge. Therefore, this study reviewed clinical data from the past 10 years to enhance clinicians’ understanding of pediatric MSOLs and improve their differential diagnosis.

## Materials and methods

Cases were identified from the database of a single institution (Children’s Hospital Affiliated to Shandong University, Jinan, China). We conducted a standardized retrospective search of the institutional database for radiology reports of either computed tomography (CT) or magnetic resonance imaging (MRI) performed between January 2015 and December 2024. Not all patients underwent both modalities; the choice of imaging was determined by clinical indication. The search included either one of the following terms: “mediastinal space-occupying lesion”, “mediastinal lesion”, “mediastinal mass”, “mediastinal tumor” or “mediastinal abnormality”. Only patients with mediastinal space-occupying diseases who had undergone imaging examinations, tissue biopsies or surgical resections and had clear diagnoses were included in the study. Patients without confirmed diagnoses were excluded.

A total of 461 confirmed cases of pediatric MSOLs were retrospectively reviewed. Clinical data on etiology, demographic characteristics, clinical manifestations, imaging features, and histopathological diagnosis were retrieved from medical records.

This study was approved by the Ethics Committee of Children’s Hospital Affiliated to Shandong University (SDFE-2024071). Informed consent was obtained from all participants’ parents. All methods were carried out in accordance with relevant guidelines and regulations.

### Statistical analysis

Statistical analyses were performed using SPSS version 17.0 (SPSS Inc., Chicago, IL, USA). Differences in the age of onset among children in different disease groups were assessed using one-way ANOVA, with pairwise comparisons conducted via Tamhane’s T2 test. P<0.05 was considered statistically significant. Figures were generated using GraphPad Prism (version 7 for Windows, GraphPad Software, La Jolla, California, USA).

## Results

### Etiology

**Figure 2:**
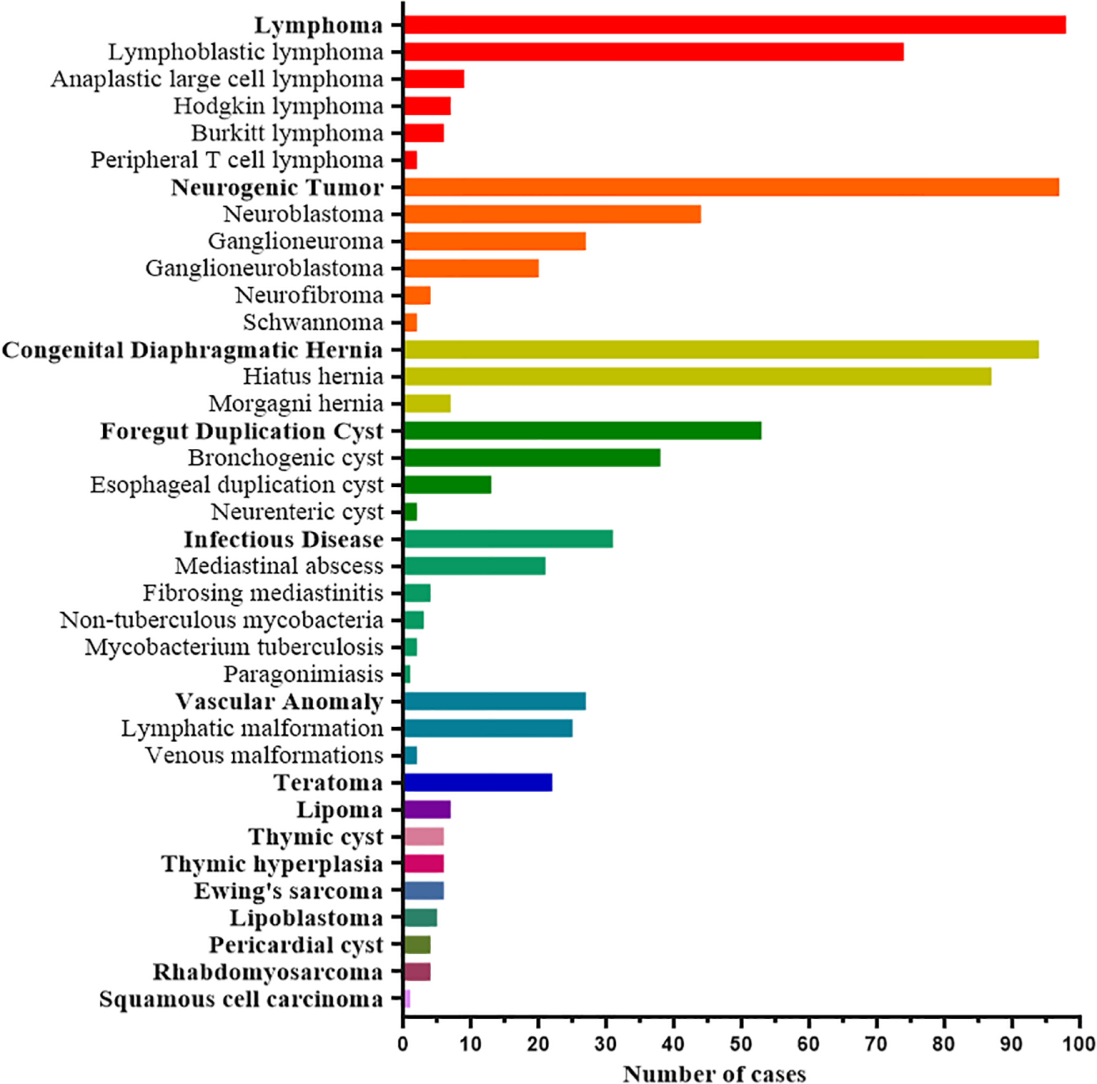
Spectrum of pediatric MSOLs in order of different categories of diseases frequency.

By disease category, the most common pediatric MSOL was lymphoma (21.3%, n=98), followed by neurogenic tumors (21.0%, n=97), CDH (20.4%, n=94), FDC (11.4%, n=53), infectious diseases (6.8%, n=31), and vascular anomalies (5.8%, n=27). By specific diagnosis, the most frequent entities were hiatus hernia (HH; 18.9%, n=87), lymphoblastic lymphoma (LBL; 16.1%, n=74), neuroblastoma (NB; 9.5%, n=44), bronchogenic cyst (BC; 8.2%, n=38), ganglioneuroma (GN; 5.9%, n=27), and lymphatic malformation (LM; 5.4%, n=25). This spectrum of pediatric MSOLs was summarized graphically in **Table 1** and **Figure 2**.

**Table 1:** Etiology of MSOLs in children.

Causes	Numbers (%)
**Lymphoma**	**98 (21.3%)**
**Non-Hodgkin Lymphoma**	**91 (19.8%)**
Lymphoblastic lymphoma	74 (16.1%)
Anaplastic large cell lymphoma	9 (2.0%)
Burkitt lymphoma	6 (1.3%)
Peripheral T cell lymphoma	2 (0.4%)
**Hodgkin lymphoma**	**7 (1.5%)**
**Neurogenic Tumor**	**97 (21%)**
Neuroblastoma	44 (9.5%)
Ganglioneuroma	27 (5.9%)
Ganglioneuroblastoma	20 (4.3%)
Neurofibroma	4 (0.9%)
Schwannoma	2 (0.4%)
**Congenital Diaphragmatic Hernia**	**94 (20.4%)**
Hiatus hernia	87 (18.9%)
Morgagni hernia	7 (1.5%)
**Foregut Duplication Cyst**	**53 (11.4%)**
Bronchogenic cyst	38 (8.2%)
Esophageal duplication cyst	13 (2.8%)
Neurenteric cyst	2 (0.4%)
**Infectious Disease**	**31 (6.8%)**
Mediastinal abscess	21 (4.6%)
Fibrosing mediastinitis	4 (0.9%)
Non-tuberculous mycobacteria	3 (0.7%)
Mycobacterium tuberculosis	2 (0.4%)
Paragonimiasis	1 (0.2%)
**Vascular Anomalies**	**27 (5.8%)**
Lymphatic malformation	25 (5.4%)
Venous malformations	2 (0.4%)
**Teratoma**	**22 (4.8%)**
**Lipoma**	**7 (1.5%)**
**Ewing's sarcoma**	**6 (1.3%)**
**Thymic hyperplasia**	**6 (1.3%)**
**Thymic cyst**	**6 (1.3%)**
**Lipoblastoma**	**5 (1.1%)**
**Rhabdomyosarcoma**	**4 (0.9%)**
**Pericardial Cyst**	**4 (0.9%)**
**Squamous cell carcinoma**	**1 (0.2%)**
**Total**	**461 (100%)**

Bold type indicates the main classification of etiology; non-bold entries are specific subtypes under each category.

### Demographic characteristics and general aspects

Basic information including age, sex, area of residence, maternal respiratory infection and maternal smoking during pregnancy among the different etiologies were shown in **Table 2**. Age was the most important initial consideration in the evaluation of a pediatric mediastinal lesion, as the prevalence of specific lesion types varied significantly across age groups.

**Table 2:** Demographic characteristics and general aspects among different causes of MSOLs in children.

Mediastinal lesion type	Numbers	Age (months)	Gender (male/female)	Area of residence (Urban/Rural)	Maternal respiratory infection during pregnancy	Maternal smoking during pregnancy
NHL	91					
T-LBL	73	75.53±42.28	49/24	16/57	6.8%	5.5%
ALCL	9	58.67±33.99	6/3	4/5	0	11.1%
BL	6	118±27.8	5/1	4/2	0	0
PTCL	2	24-54	2/0	1/1	50%	0
B-LBL	1	120	0/1	0/1	100%	0
**HL**	7	138.9±15.27	4/3	5/2	14.3%	0
Neurogenic Tumor	97					
NB	44	31.22±28.25	26/18	24/20	6.8%	0
GN	27	72.37±23.22	17/10	15/12	0	7.4%
GNB	20	66.5±28.56	9/11	12/8	10%	0
NF	4	41-108	2/2	2/2	0	25%
Schwannoma	2	96-120	1/1	2/0	0	0
CDH	94					
HH	87	23.38±33.30	55/32	27/60	5.7%	4.6%
MH	7	29.86±27.37	3/4	2/5	0	0
**FDC**	53					
BC	38	50.13±37.51	21/17	25/13	10.5%	5.3%
EDC	13	65.80±59.74	5/8	9/4	0	23.1%
NC	2	18-24	1/1	1/1	50%	0
Infectious Disease	31					
MA	21	44.57±38.37	12/9	9/12	9.5%	0
FM	4	18-42	2/2	3/1	0	0
NTM	3	24-38	2/1	2/1	33.3%	0
MTB	2	13-20	2/0	2/0	0	0
Paragonimiasis	1	156	0/1	0/1	0	0
Vascular Anomalies	27					
LM	25	28.82±36.89	13/12	14/11	12%	0
VM	2	35-84	1/1	1/1	0	50%
Teratoma	22	62.87±46.49	13/9	8/14	9%	0
Lipoma	7	82.14±36.25	6/1	5/2	0	0
ES	6	57.33±22.41	4/2	3/3	0	0
TH	6	22.67±26.95	5/1	4/2	0	33.3%
TC	6	33.38±61.09	2/4	5/1	16.7%	0
Lipoblastoma	5	12-34	3/2	1/4	0	20%
RMS	4	12-132	2/2	2/2	0	0
PC	4	32-45	3/1	3/1	50%	0
SCC	1	180	0/1	0/1	100%	0

NHL, Non-Hodgkin Lymphoma; LBL, Lymphoblastic lymphoma; ALCL, Anaplastic large cell lymphoma; BL, Burkitt lymphoma; PTCL, Peripheral T cell lymphoma; HL, Hodgkin lymphoma; NB, Neuroblastoma; GN, Ganglioneuroma; GNB, Ganglioneuroblastoma; NF, Neurofibroma; CDH, Congenital Diaphragmatic Hernia; HH, Hiatus hernia; MH, Morgagni hernia; FDC, Foregut Duplication Cyst; BC, Bronchogenic cyst; EDC, Esophageal duplication cyst; NC, Neurenteric cyst; MA, Mediastinal abscess; FM, Fibrosing mediastinitis; NTM, Non-tuberculous mycobacteria; MTB, Mycobacterium tuberculosis; LM, Lymphatic malformation; VM, Venous malformation; ES, Ewing's sarcom; TH, Thymic hyperplasia; TC, Thymic cyst; RMS, Rhabdomyosarcoma; PC, Pericardial Cyst; SCC, Squamous cell carcinoma.

Bold font in the first column denotes the main category of mediastinal lesions, with the corresponding total number of cases shown in the adjacent "Numbers" column. Subtypes under each category are listed in regular font with their respective case numbers.

**Figure 3:**
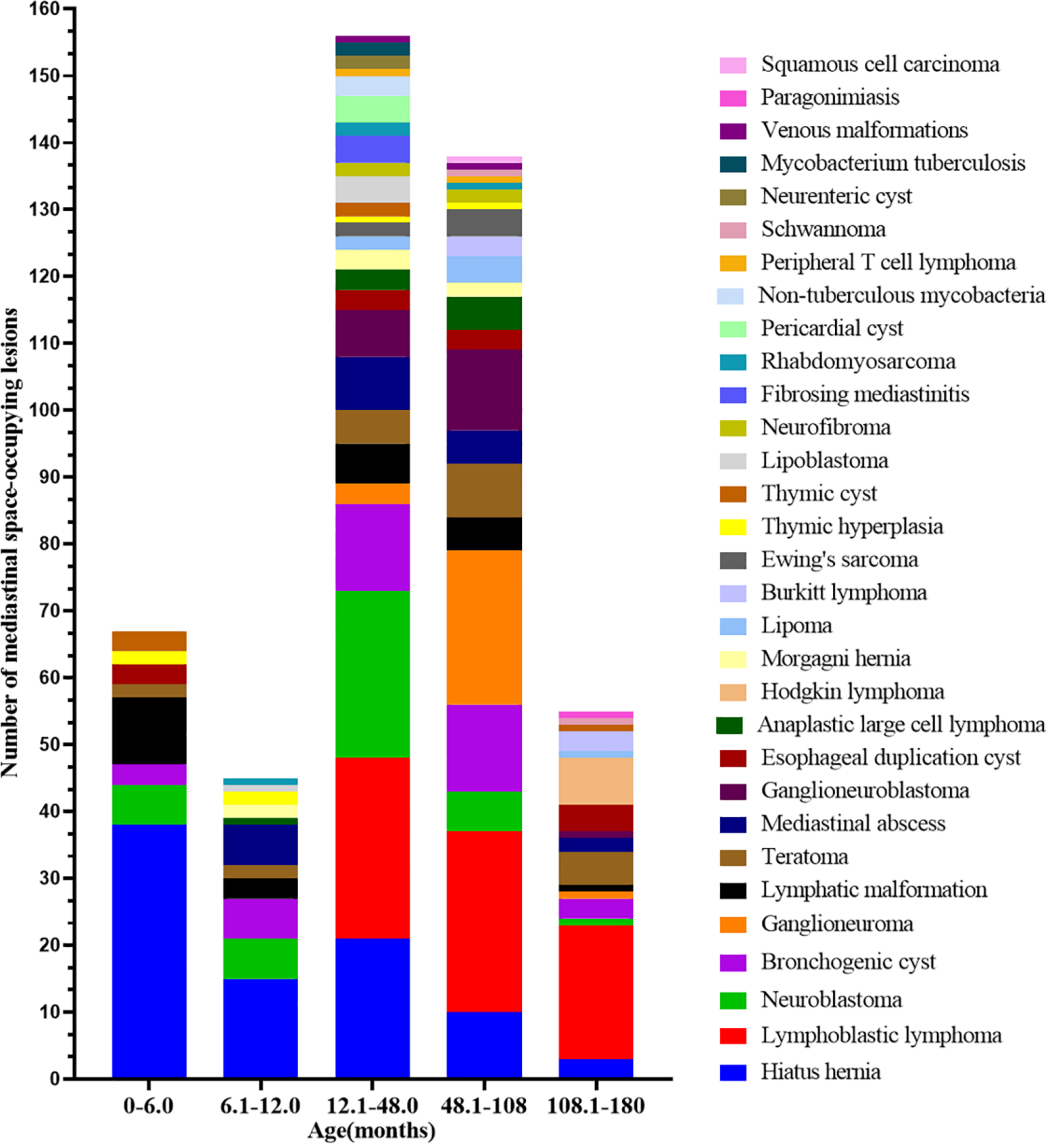
The frequency of mediastinal space-occupying lesions by months of age.

The etiologies of MSOLs in children of different age groups were shown in **Figure 3**. In infants aged 0–6 months, HH, LM, and NB were most common; at 6–12 months, HH, NB, and BC predominated. Among children aged 12–48 months, LBL, NB, and HH were frequent; at 48–108 months, LBL, GN, and ganglioneuroblastoma (GNB) prevailed; and in those aged 108–180 months, LBL, peripheral T cell lymphoma (PTCL), and teratoma were the leading entities.This descriptive age-related distribution was further supported by statistical comparison (**Table 3**). Patients with CDH were the youngest, significantly younger than those with lymphoma, neurogenic tumors, foregut duplication cysts, and other tumors (P < 0.01). Patients with lymphoma were significantly older than those with CDH, foregut duplication cysts, and infectious diseases (P < 0.01). No significant age differences were detected among neurogenic tumors, foregut duplication cysts, infectious diseases, vascular anomalies, and other tumors, indicating substantial overlap in age distribution among these entities.

**Table 3:** Comparison of the age of onset among children in different disease groups.

Disease groups	Lymphoma	Neurogenic tumor	CDH	FDC	Infectious diseases	Vascular anomaly	Other tumors	Others
Lymphoma (N=98)	—	**<**0.01 (10.10,45.50)	**<**0.01 (39.30,74.60)	**<**0.01 (39.30,74.60)	**<**0.01 (10.76,64.93)	**<**0.01 (22.05,77.41)	0.47 (-7.23,44.95)	**<**0.01 (12.05,88.42)
Neurogenic tumor (N=97)	**<**0.01 (-45.50,-10.10)	—	<0.01 (13.96,44.35)	0.98 (-22.08,23.35)	0.99 (-15.71,35.81)	0.21 (-4.50,48.35)	0.90 (-33.57,15.69)	0.71 (-15.17,60.03)
CDH (N=94)	**<**0.01 (-74.60,-39.30)	**<**0.01 (-44.35,-13.96,)	—	**<**0.01 (-51.19,-6.84)	0.40 (-44.84,6.62)	0.23 (-33.62,19.17)	**<**0.01 (-62.69,13.50)	0.53 (-44.31,30.87)
FDC (N=53)	**<**0.01 (-51.80,-4.08)	0.98 (-23.35,22.08)	**<**0.01 (6.84,51.19)	—	0.26 (-19.70,39.79)	0.47 (-8.58,52.15)	0.25 (-28.15,19.99)	0.84 (-17.38,61.97)
Infectious diseases (N=31)	**<**0.01 (-64.93,-10.76)	0.99 (-35.81,15.71)	0.40 (-6.62,44.84)	0.87 (-39.79,19.97)	—	0.25 (-20.79,44.55)	0.80 (-50.53,12.55)	0.34 (-28.73,53.51)
Vascular anomaly (N=27)	**<**0.01 (-77.41,-22.05)	0.21 (-48.35,4.50)	0.23 (-19.17,33.62)	0.47 (-52.15,8.58)	0.25 (-44.55,20.79)	—	0.07 (-62.85,1.12)	0.12 (-40.88,41.90)
Other tumors (N=45)	0.47 (-44.95,7.23)	0.90 (-15.69.33.57)	**<**0.01 (-13.50,62.69)	0.25 (-19.99,28.15)	0.80 (-12.55,50.53)	0.07 (-1.12,62.85)	—	0.31 (-9.30,72.06)
Others (N=16)	**<**0.01 (-88.42,-12.05)	0.71 (-60.03,15.17)	0.53 (-30.87,44.31)	0.84 (-61.97,17.38)	0.34 (-53.51,27.83)	0.12 (-41.90,40.88)	0.31 (-72.06,9.30)	—

1.The values in the table are p-values (95% confidence interval), with p<0.05, indicating a statistically significant difference.

2. Lymphoma includes Hodgkin's lymphoma and non-Hodgkin's lymphoma. Neurogenic tumors include Neuroblastoma, Ganglioneuroma, Ganglioneuroblastoma, Neurofibroma and Schwannoma. Congenital diaphragmatic hernias include Morgagni hernia and Hiatus herniaa; Foregut Duplication Cyst include Bronchogenic cyst, Esophageal duplication cyst, and Neurenteric cyst. Infectious diseases include mediastinal abscess, Fibrosing mediastinitis, Non-tuberculous mycobacteria, Mycobacterium tuberculosis and Paragonimiasis. Vascular anomaly include Lymphatic malformation and Venous malformation. Other tumors include Teratoma, Ewing's sarcoma, Lipoma, Lipoblastoma and Rhabdomyosarcoma. Others include Thymic hyperplasia, Thymic cysts and Pericardial Cyst.

CDH=Congenital Diaphragmatic Hernia; FDC=Foregut Duplication Cyst.

Some other general characteristics were also observed in **Table 2**. For HH, the male-to-female ratio was 55:32, the urban-to-rural ratio was 27:60, the respiratory infection rate during pregnancy was 5.7%, and the smoking rate during pregnancy was 4.6%; for LBL, the male-to-female ratio was 49:24, the urban-to-rural ratio was 16:57, the respiratory infection rate during pregnancy was 6.8%, and the smoking rate during pregnancy was 5.5%; for NB, the male-to-female ratio was 26:18, the urban-to-rural ratio was 24:20, the respiratory infection rate during pregnancy was 6.8%, and the smoking rate during pregnancy was 0%, respectively.

### The mediastinal localization of the MSOLs

The localization of the mediastinal space-occupying lesions was shown in **Table 4**. Most lesions were found to reside within a single mediastinal compartment. Of 461 lesions, 180 (39%) were located in the prevascular mediastinum, followed by 137 (29.7%) in the visceral mediastinum and 104 (22.6%) in the paravertebral mediastinum respectively. In 40 (8.7%) patients, the lesions involved multiple compartments. Among lesions spanning more than one compartment, the majority involved both the visceral and paravertebral compartments (27 of 461), followed by lesions in the prevascular, visceral and paravertebral (7), and prevascular and visceral (6) compartments.

**Table 4:** The mediastinal space-occupying lesion localization.

Compartment	Lesions	Numbers (%)
Prevascular compartment	T-lymphoblastic lymphoma	72 (40%)
	Teratoma	22 (12.2%)
	Lymphatic malformation	16 (8.9%)
	Mediastinal abscess	10 (5.6%)
	Morgagni hernia	7 (3.9%)
	Hodgkin lymphoma	7 (3.9%)
	Lipoma	7 (3.9%)
	Thymic hyperplasia	6 (3.3%)
	Thymic cyst	6 (3.3%)
	Burkitt lymphoma	6 (3.3%)
	Anaplastic large cell lymphoma	6 (3.3%)
	Lipoblastoma	5 (2.8%)
	Venous malformations	2 (1.1%)
	Ewing's sarcoma	2 (1.1%)
	Rhabdomyosarcoma	2 (1.1%)
	Peripheral T cell lymphoma	2 (1.1%)
	Schistosoma granuloma	1 (0.6%)
	Esophageal duplication cyst	1 (0.6%)
Visceral compartment	Hiatus hernia	69 (50.4%)
	Bronchogenic cyst	26 (19.0%)
	Esophageal duplication cyst	11 (8.0%)
	Mediastinal abscess	10 (7.3%)
	Lymphatic malformation	7 (5.1%)
	Fibrosing mediastinitis	4 (2.9%)
	Pericardial Cyst	4 (2.9%)
	Non-tuberculous mycobacteria	2 (1.5%)
	Rhabdomyosarcoma	2 (1.5%)
	Anaplastic large cell lymphoma	1 (0.7%)
	Squamous cell carcinoma	1 (0.7%)
Paravertebral compartment	Neuroblastoma	38 (36.5%)
	Ganglioneuroma	22 (21.2%)
	Ganglioneuroblastoma	20 (19.2%)
	Bronchogenic cyst	10 (9.6%)
	Neurofibroma	4 (3.8%)
	Ewing's sarcoma	4 (3.8%)
	Schwannoma	2 (1.9%)
	B-lymphoblastic lymphoma	1 (1.0%)
	Anaplastic large cell lymphoma	1 (1.0%)
	Esophageal duplication cyst	1 (1.0%)
	Lymphatic malformation	1 (1.0%)
Multiple compartments	Hiatus hernia	18 (45.0%)
	Neuroblastoma	6 (15.0%)
	Ganglioneuroma	5 (12.5%)
	Mycobacterium tuberculosis	2 (5.0%)
	Bronchogenic cyst	2 (5.0%)
	Neurenteric cyst	2 (5.0%)
	Mediastinal abscess	1 (2.5%)
	T-Lymphoblastic lymphoma	1 (2.5%)
	Non-tuberculous mycobacteria	1 (2.5%)
	Anaplastic large cell lymphoma	1 (2.5%)
	Lymphatic malformation	1 (2.5%)

**Figure 4:**
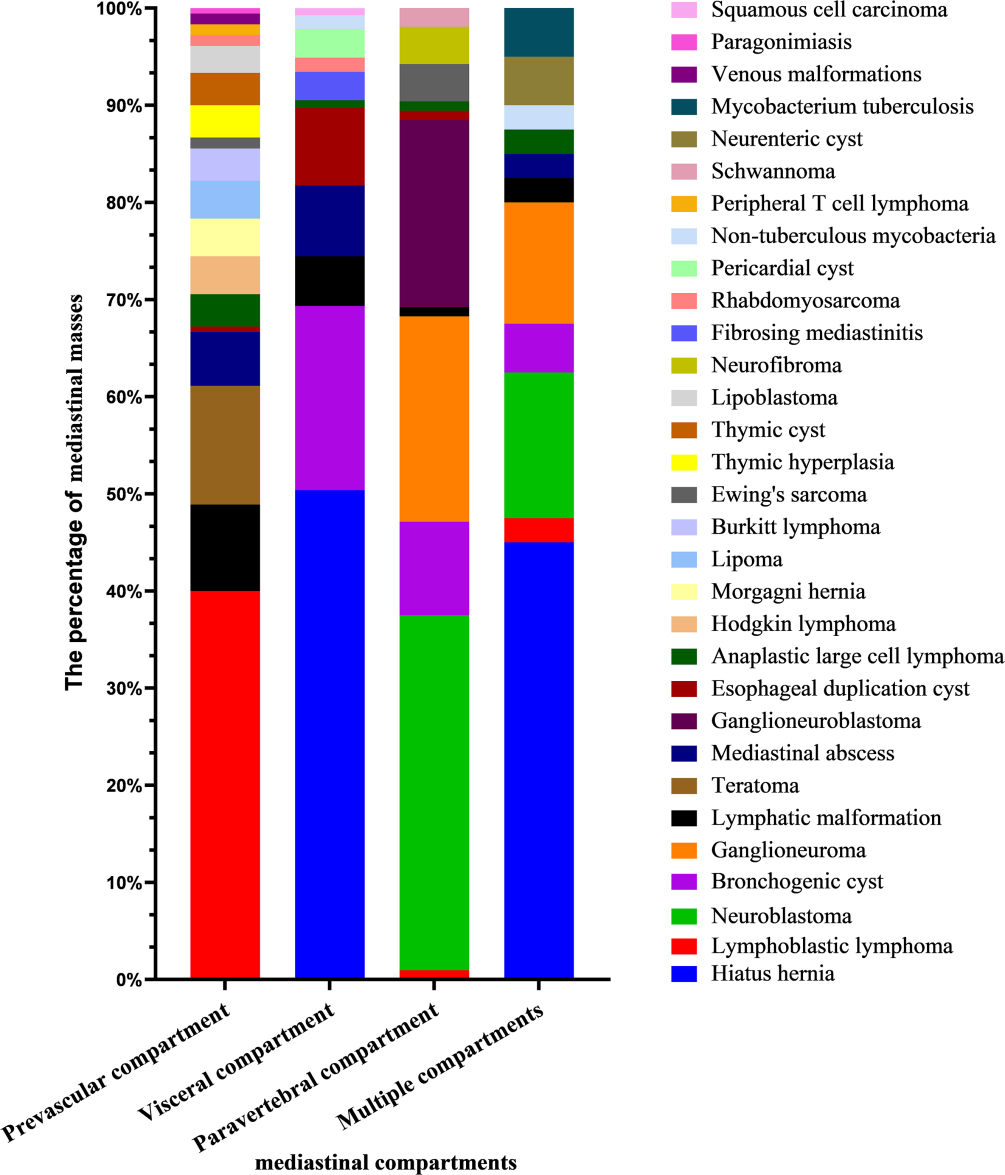
The percentage of MSOLs in the same mediastinum.

The frequency of mediastinal lesions based on mediastinal compartment was summarized in **Figure 4**. In the prevascular mediastinum, T-LBL (40.0%) was the most common lesion, followed by teratoma (12.2%) and LM (8.9%). In the visceral mediastinum, HH (50.4%) was the predominant lesion, followed by BC (19.0%) and esophageal duplication cyst (EDC; 8.0%). In the paravertebral mediastinum, NB (36.5%) was the most often identified lesion, followed by GN (21.2%) and GNB (19.2%).

### Clinical manifestations

All clinical manifestations reported in the selected studies and their frequencies are shown in **Table 5**. Forty patients were asymptomatic. The most common symptom was cough (53.4%), followed by chest oppression (21.3%), fever (19.5%), nausea/vomiting (13.9%), chest pain (12.1%), facial or neck edema (7.4%), localized pain (6.9%), dyspnea (6.5%), fatigue (3.7%), bilateral lower extremity weakness (1.7%), and gait disturbance (1.5%).

**Table 5:** Distributions of clinical manifestations of children with MSOLs described in the selected studies.

Disease	Asymptomatic	Fever	Cough	Chest pain	Chest oppression	Dyspnea	Nausea /vomiting	Body part pain	Bilateral lower extremity weakness	Gait disturbance	Fatigue	Cervicofacial edema
T-LBL	3	8	49	33	52	11	2	9	0	0	3	13
ALCL	1	4	5	0	2	1	0	0	0	0	0	2
BL	0	0	3	0	0	0	1	3	0	0	1	1
PTCL	0	1	1	0	1	0	0	0	0	0	0	0
B-LBL	0	0	0	0	0	0	0	0	0	0	0	1
HL	0	0	2	2	1	0	0	0	0	0	1	2
NB	4	8	26	5	9	5	2	9	5	6	7	1
GN	5	6	18	3	4	1	0	3	1	0	1	1
GNB	1	5	13	1	2	1	2	0	0	0	0	0
NF	0	0	4	0	0	0	0	0	0	0	0	0
Schwannoma	0	0	0	0	0	0	0	0	0	1	1	0
HH	6	7	21	2	4	0	55	2	0	0	0	0
MH	1	2	6	0	1	0	0	0	0	0	0	0
BC	7	10	19	0	6	2	0	1	0	0	1	0
EDC	2	8	9	0	0	0	1	0	0	0	0	1
NC	0	0	2	0	0	0	0	0	0	0	0	0
MA	0	14	17	3	3	1	0	0	0	0	0	1
FM	0	2	4	0	0	0	0	0	0	0	0	0
NTM	0	3	2	0	0	0	0	0	0	0	0	0
Mtb	0	2	0	0	0	0	0	0	0	0	0	0
Paragonimiasis	0	0	1	0	0	0	0	0	0	0	0	0
LM	3	0	14	1	3	0	0	2	0	0	2	12
VM	0	0	2	0	0	0	0	0	0	0	0	0
Teratoma	1	5	10	3	2	0	0	2	0	0	0	0
Lipoma	3	2	3	0	0	0	1	0	0	0	0	0
ES	0	0	1	0	2	1	0	1	2	0	0	0
TH	1	0	4	0	2	0	0	0	0	0	0	0
TC	2	0	3	2	0	0	0	0	0	0	0	0
Lipoblastoma	0	0	1	0	1	4	0	0	0	0	0	0
RMS	0	0	2	1	1	2	0	0	0	0	0	0
PC	0	2	3	0	1	1	0	0	0	0	0	0
SCC	0	1	1	0	1	0	0	0	0	0	0	0

Lymphoblastic lymphoma;ALCL, Anaplastic large cell lymphoma;BL, Burkitt lymphoma;PTCL, Peripheral T cell lymphoma; HL, Hodgkin lymphoma; NB, Neuroblastoma;GN, Ganglioneuroma;GNB, Ganglioneuroblastoma;NF, Neurofibroma; CDH, Congenital Diaphragmatic Hernia; HH, Hiatus hernia;MH, Morgagni hernia, FDC, Foregut Duplication Cyst;BC, Bronchogenic cyst;EDC, Esophageal duplication cyst, NC, Neurenteric cyst; MA, Mediastinal abscess;FM, Fibrosing mediastinitis; NTM, Non-tuberculous mycobacteria; MTB, Mycobacterium tuberculosis;LM, Lymphatic malformation; VM, Venous malformation; ES, Ewing's sarcom;TH, Thymic hyperplasia;TC, Thymic cyst;RMS, Rhabdomyosarcoma; PC, Pericardial Cyst; SCC, Squamous cell carcinoma.

Despite all being MSOLs, the clinical manifestations of each disease varied significantly. Lymphoma primarily presented with chest oppression, cough, and chest pain, while nausea/vomiting and fatigue were less common. Neurogenic tumors typically presented with cough and fever, with gait disturbance and fatigue being less common. CDH most commonly presented with nausea/vomiting and cough, while chest pain and abdominal pain were less typical. FDCs most frequently presented with cough and fever, whereas nausea/vomiting and fatigue were uncommon. Infectious diseases commonly presented with cough and fever, with dyspnea and cervicofacial edema occurring less frequently.

### Diagnostic approaches

A multimodal diagnostic strategyas was shown in **Table 6**. Among the 461 patients, a definitive diagnosis was achieved through imaging studies in 139 cases (30.2%), enabling subsequent effective treatment. This group included 94 cases of CDH diagnosed by upper gastrointestinal contrast studies, 24 cases of vascular anomalies identified via contrast-enhanced CT or MRI, and 21 cases of MA confirmed by thoracic ultrasound or contrast-enhanced CT. The remaining patients underwent surgical interventions with tissue sampling for diagnostic purposes. The types of surgical procedures performed, based on lesion characteristics, were detailed in **Table 6**. Thoracoscopic surgery was performed in 189 patients (41%), including 87 neurogenic tumors, 52 FDCs, 18 teratomas, 7 lipomas, 6 thymic hyperplasia (TH), 6 thymic cyst (TC), 5 lipoblastomas, 4 pericardial cyst (PC), 3 vascular anomalies, and 1 case of paragonimiasis.

**Table 6:** Types of diagnostic procedures performed according to lesion characteristic.

Diagnostic approaches	Numbers (%)
Thoracoscopic surgery	189 (41%)
EBUS-TBNA	17 (3.7%)
EUS-FNA	1 (0.2%)
US-PTNB	82 (17.8%)
US-PNB	28 (6.1%)
CT-PTNB	1 (0.2%)
open surgery	4 (0.9%)
Imaging studies	139 (30.2%)

EBUS-TBNA, endobronchial ultrasound-guided transbronchial needle aspiration.

EUS-FNA, endoscopic ultrasound-guided fine needle aspiration.

US-PTNB, Ultrasound-guided percutaneous transthoracic needle biopsy.

US-PNB, Ultrasound-guided percutaneous core needle biopsy.

CT-PTNB, Computed tomography guided percutaneous transthoracic needle biopsy.

Among other diagnostic procedures, ultrasound-guided percutaneous transthoracic needle biopsy was performed in 82 cases (17.8%), leading to the diagnosis of 62 lymphomas, 10 neurogenic tumors, 4 rhabdomyosarcoma (RMS), 4 teratomas, and 2 Ewing’s sarcoma (ES). Additional techniques included ultrasound-guided percutaneous core needle biopsy (6.1%), which diagnosed 28 lymphomas; endobronchial ultrasound-guided transbronchial needle aspiration (3.7%), which identified 7 lymphomas, 4 fibrosing mediastinitis, 3 non-tuberculous mycobacteria (NTM) infections, 2 MTB infections, and 1 squamous cell carcinoma (SCC); and open surgical biopsy (0.9%), which confirmed 4 ES. The remaining 2 patients underwent either endoscopic ultrasound-guided fine needle aspiration or CT-guided percutaneous transthoracic needle biopsy. No major complications, such as major bleeding, severe heart failure, severe respiratory failure, or death, were associated with the surgical or interventional procedures.

### Comparison between malignant and non-neoplastic MSOLs

A comparative analysis delineating malignant tumors (n=177, 38.4%) from non-neoplastic diseases (n=284, 61.6%) was presented in **Table 7**. Lymphoma (55.4%) and neurogenic tumors (36.2%) were the most common malignancies, while CDH (33.1%) and FDC (18.7%) were most common among non-neoplastic diseases. Malignant cases presented at an older median age (55 months *vs*. 34 months), were primarily located in the prevascular (63.9%) and paravertebral (61.5%) compartments, and frequently manifested with chest oppression (74.5%) or pain (80.4%). In contrast, non-neoplastic lesions overwhelmingly involved the visceral compartment (97.1%), more commonly presented with nausea/vomiting (89.1%) or were asymptomatic (75% of asymptomatic cases). Diagnostic pathways diverged substantially. All cases diagnosed by imaging alone (n=139) are non-neoplastic. Image-guided biopsy was the primary diagnostic method for malignancies (67.2%), whereas surgical resection was more commonly employed for non-neoplastic diseases (59.2%).

**Table 7:** Comparative analysis of malignant versus non-neoplastic MSOLs.

Parameter	Malignant tumors	Non-neoplastic diseases
Number of cases (%)	177(38.4%)	284(61.6%)
Most common diagnosis(top3)	Lymphoma(98,55.4%)	CDH (94,33.1%)
	Neurogenic Tumor(64,36.2%)	FDC (53,18.7%)
	Ewing's sarcoma(6,3.4%)	Infectious Disease(31,10.9%)
Median age (months)	55	34
Gender (Male:Female)	109:68	155:129
Urban : Rural	72:105	133::151
Mediastinal compartment(%)		
Prevascular	115/180 (63.9%)	65/180 (36.1%)
Visceral	4/137 (2.9%)	133/137 (97.1%)
Paravertebral	64/104 (61.5%)	40/104 (38.5%)
Multiple compartments	8/40 (20%)	32/40 (80%)
Common symptoms		
Cough	113/246 (45.9%)	133/246 (54.1%)
Chest oppression	73/98 (74.5%)	25/98 (25.5%)
Fever	32/90 (35.6%)	58/90 (64.4)
Nausea/Vomiting	7/64 (10.9%)	57/64 (89.1%)
Chest pain	45/56 (80.4%)	11/56 (19.6%)
Asymptomatic	10/40 (25%)	30/40 (75%)
Diagnostic approach		
Imaging only	0	139/284 (48.9%)
Image-guided biopsy	119/177 (67.2%)	10/284 (3.5%)
surgery	25/177 (14.1%)	168/284 (59.2%)

## Discussion

This large, single-center retrospective study of 461 pediatric patients provides a comprehensive analysis of the clinical spectrum of MSOLs in children. Our findings delineate a distinct etiological profile, underscore the paramount importance of age and lesion localization in differential diagnosis, and validate the efficacy and safety of a contemporary, multimodal diagnostic strategy. Furthermore, the comparative analysis between malignant and non-neoplastic lesions provides a refined clinical framework, highlighting distinct demographic, anatomical, and diagnostic pathways that directly inform management decisions. This offers significant insights that both confirm and refine existing paradigms in pediatric MSOL management.

Despite its descriptive nature, this study offers several novel contributions that extend beyond the existing pediatric MSOLs literature. First, as the largest pediatric MSOL cohort from China, it demonstrates a distinct epidemiological profile. Second, we provide the first comprehensive head-to-head comparison between malignant and non-neoplastic MSOLs (**Table 7**), integrating age, compartment, symptoms, and diagnostic pathways into a unified framework. Third, we contribute large-scale data on minimally invasive diagnostic techniques in children. With 82 ultrasound-guided percutaneous transthoracic needle biopsy (US-PTNB) and 17 endobronchial ultrasound-guided transbronchial needle aspiration (EBUS-TBNA), this represents one of the largest pediatric validation cohorts to date. We also found that 30.2% of cases were diagnosed by imaging alone, thereby avoiding unnecessary biopsies.

One of the most salient findings of our study is the etiological spectrum, where lymphoma emerged as the most common MSOLs, closely followed by neurogenic tumors and CDH. Our findings delineate a distinctive etiological triad, with lymphoma (21.3%), neurogenic tumors (21.0%), and CDH (20.4%) constituting remarkably similar and predominant proportions. This profile underscores that the differential diagnosis for a pediatric mediastinal mass must broadly encompass malignant, benign, and congenital entities. The high frequency of CDH, predominantly HH, highlights a critical consideration in the Chinese pediatric population, suggesting that congenital anomalies constitute a substantial portion of mediastinal pathologies, a factor that must be prominently integrated into the differential diagnosis, particularly in infants. This spectrum aligns with more recent cohorts that report a significant proportion of non-neoplastic lesions (10, 11), reminding clinicians that not every mediastinal mass is a tumor—a distinction that carries direct implications for the urgency of intervention and follow-up strategy.

Our data elucidate a clear age-dependent distribution of MSOLs. The predominance of HH and LM in infants (<12 months) contrasts sharply with the peak incidence of LBL in children aged 1–15 years. This pattern is consistent with the natural history of these diseases—congenital lesions presenting early and malignancies like lymphoma having a later onset. This age-stratification is a fundamental clinical tool that, when combined with compartmental localization, creates a powerful algorithm for refining diagnostic possibilities and prioritizing interventions based on malignant potential (9, 12–15). For example, recognizing that a mediastinal mass in an older child is more likely to be lymphoma can accelerate referral to pediatric oncology, thereby reducing diagnostic delay and enabling earlier initiation of therapy—a factor known to improve outcomes in aggressive malignancies such as lymphoblastic lymphoma.

Furthermore, our results powerfully reinforce the well-established yet crucial principle of compartment-specific predilection, as defined by the ITMIG classification. The strong association of T-LBL with the prevascular compartment, HH with the visceral compartment, and NB with the paravertebral compartment provides an indispensable initial diagnostic framework. This compartmental predilection was markedly different between malignant and non-neoplastic groups. Malignant tumors predominantly occupied the prevascular and paravertebral compartments, whereas non-neoplastic lesions overwhelmingly involved the visceral compartment. This anatomically stratified approach, consistent with contemporary radiological literature (2, 4), allows for a significantly refined differential diagnosis upon initial imaging, thereby guiding subsequent diagnostic steps more efficiently and shortening the time to definitive diagnosis. For instance, a prevascular mass in an older child immediately raises suspicion for lymphoma (16, 17), prompting hematologic work-up and consideration of minimally invasive biopsy, while a paravertebral mass in a toddler strongly suggests a neurogenic tumor (18–20), which may require surgical planning for complete resection given its potential for malignant behavior. Such localization-driven triage directly impacts management pathways and can mitigate risks associated with delayed diagnosis, such as spinal cord compression in paravertebral tumors or superior vena cava syndrome in anterior mediastinal masses.

The clinical presentation was heterogeneous, with 8.7% of patients being asymptomatic, diagnosed incidentally —a figure that underscores the value of imaging for unrelated indications. Cough was the most common symptom, likely due to the mass effect on the tracheobronchial tree. However, the symptom profile was disease-specific. The high frequency of nausea/vomiting in CDH, cervicofacial edema in lymphoma (indicative of superior vena cava syndrome), and neurologic symptoms in posterior mediastinal tumors provides key clues for clinical suspicion.Our comparative analysis further clarified this distinction: symptoms suggestive of compression or invasion (chest oppression, pain) were strongly associated with malignancies, while gastrointestinal symptoms (nausea/vomiting) and asymptomatic presentation were more characteristic of non-neoplastic conditions.Recognizing these symptomatic patterns is crucial not only for differential diagnosis but also for anticipating complications and tailoring urgent interventions. For instance, the presence of superior vena cava syndrome in lymphoma necessitates expedited diagnostic steps and potential emergent therapy to avoid life-threatening complications, directly linking symptom recognition to prognosis.

A cornerstone of our study is the detailed analysis of the diagnostic pathway. We demonstrated that a successful diagnostic strategy is inherently multimodal. A significant proportion of cases (30.2%), including most CDHs and vascular anomalies, were definitively diagnosed through non-invasive imaging alone (e.g., upper GI series, contrast-enhanced CT/MRI), effectively avoiding the risks of surgical or interventional procedures. This highlights the pivotal role of expert radiological interpretation. This non-invasive diagnostic success was almost exclusively within the non-neoplastic group, underscoring that a substantial portion of these lesions can be managed without biopsy —thereby eliminating procedure-related delays and risks, and allowing for timely conservative or surgical management as indicated. For lesions requiring tissue diagnosis, our data reflect a pronounced shift towards minimally invasive techniques over the past decade. The high utilization and success rates of thoracoscopic surgery (41%) and various image-guided percutaneous biopsies (e.g., US-PTNB 17.8%, EBUS-TBNA 3.7%) are noteworthy. This trend is supported by a growing body of literature which affirms the diagnostic yield and safety of these techniques in children (21–24). Importantly, the adoption of these minimally invasive techniques facilitates quicker pathological diagnosis, which is critical for malignancies where treatment initiation delay can adversely affect prognosis. In our cohort, the use of image-guided biopsy as the primary diagnostic method for malignancies (67.2%) enabled rapid histopathological confirmation.

When interpreting our findings, several limitations must be considered. The retrospective, single-center design, while providing a large sample, may limit the generalizability of our specific etiological spectrum to other geographic regions or healthcare settings. Furthermore, the study includes only patients with a definitive diagnosis, potentially introducing a selection bias by excluding complex cases that remained undiagnosed or were managed conservatively without a pathological confirmation.

In conclusion, our study delineates the diverse, age-dependent, and compartment-specific landscape of pediatric MSOLs in a large Chinese cohort. Synthesizing these findings, we propose a practical triage framework that integrates four readily accessible parameters—age, compartment, symptom pattern, and diagnostic pathway—to guide early clinical decision-making. Specifically, an older child with a prevascular/paravertebral mass and chest symptoms should be prioritized for malignancy work-up (urgent oncology referral and biopsy), whereas an infant with a visceral lesion and gastrointestinal or no symptoms can be managed with an imaging-first approach. This framework shifts the diagnostic paradigm from descriptive categorization to proactive, risk-stratified triage, directly informing referral urgency and the choice between non-invasive and invasive pathways.

## Data Availability

The original contributions presented in the study are included in the article/supplementary material. Further inquiries can be directed to the corresponding author.
